# PBIP: a deep learning framework for predicting phage–bacterium interactions at the strain level

**DOI:** 10.1093/bib/bbaf656

**Published:** 2025-12-10

**Authors:** Lijia Ma, Peng Gao, Gufeng Liu, Yuan Bai, Qiuzhen Lin, Jianqiang Li, Minfeng Xiao

**Affiliations:** College of Computer Science and Software Engineering, Shenzhen University, No. 3688 Nanhai Avenue, Nanshan District, Shenzhen 518060, Guangdong, China; College of Computer Science and Software Engineering, Shenzhen University, No. 3688 Nanhai Avenue, Nanshan District, Shenzhen 518060, Guangdong, China; College of Computer Science and Software Engineering, Shenzhen University, No. 3688 Nanhai Avenue, Nanshan District, Shenzhen 518060, Guangdong, China; School of Public Health, The University of Hong Kong, No. 7 Sassoon Road, Pokfulam, Hong Kong SAR, China; College of Computer Science and Software Engineering, Shenzhen University, No. 3688 Nanhai Avenue, Nanshan District, Shenzhen 518060, Guangdong, China; National Engineering Laboratory for Big Data System Computing Technology, Shenzhen University, No. 3688 Nanhai Avenue, Nanshan District, Shenzhen 518060, Guangdong, China; BGI-Shenzhen, Beishan Industrial Zone, Yantian District, Shenzhen 518083, Guangdong, China

**Keywords:** deep learning, protein representation learning, phage–bacterium interactions, attention mechanism

## Abstract

Phage therapy has received great attention as a promising antimicrobial treatment, and its core technique, namely predicting phage–bacterium interactions (PBIs), is crucial for understanding infection mechanisms and optimizing therapeutic strategies. However, existing computational methods mainly focus on the species or higher taxonomic levels, and usually neglect the potential of deep embedding representations, limiting their ability to capture complex biological patterns inherent in sequences. This hinders the discovery of rich sequence features, and restricts the clinical application of phage therapy. To address these limitations, we propose a novel deep learning framework (called PBIP) for strain-level PBI prediction. In PBIP, we first identify strain-level interactions through biological infection experiments and sequencing of Klebsiella pneumoniae isolated from the clinical environment of Xiangya Hospital. Then, we utilize a pretrained unified representation model to convert protein sequences of phages and bacteria into deep embeddings. Next, we apply the synthetic minority oversampling technique to generate positive interactions in the embedding space to address the data imbalance issue. Subsequently, we design a deep neural network that uses a convolutional neural network to extract local features, a bi-directional gated recurrent unit to capture global features, and an attention module to highlight significant features. Finally, a fully connected layer integrates this information for PBI prediction. Experimental results show the superiority of PBIP over the state-of-the-art methods in predicting PBIs. The code and datasets are available at https://github.com/a1678019300/PBIP.

## Introduction

Phages are viruses that infect bacteria and replicate within them [1, 2], playing vital roles in regulating bacterial populations and maintaining ecological balance in various environments [3]. Moreover, the potential of phages as an alternative to antibiotics has gained increasing attention, forming the basis of phage therapy [4, 5]. The key step in implementing phage therapy is to identify effective phages that can interact with specific bacterial hosts and lyse them. Therefore, predicting phage–bacterium interactions (PBIs), especially at the strain level, is crucial for targeted therapeutic applications [6]. However, traditional experimental approaches for verifying PBIs are time-consuming and costly, which has prompted the development of computational methods to improve efficiency.

The existing computational methods for PBI prediction are mainly categorized into alignment-based and learning-based methods. The alignment-based methods predict PBIs by assessing the similarity between phages and bacteria. For example, HostPhinder [7] predicts PBIs based on $k$-mer similarity between the query phages and known host phages, while VPF-Class [8] estimates similarity by using viral protein families. Moreover, VirHostMatcher [9] predicts interaction likelihood by analyzing oligonucleotide frequency similarity between phages and bacteria, while PHIST [10] employs $k$-mer comparison to evaluate genomic similarity between phages and potential bacterial hosts. Additionally, certain methods [11–13] leverage the CRISPR system for interaction prediction, but these methods are only suitable for a subset of bacteria.

The learning-based methods utilize machine learning and deep learning to model complex PBIs. For instance, WIsH [14] trains Markov models for the candidate hosts to estimate the likelihood of their interactions with phages, while Leite $\textit{et}\ \textit{al}.$ [15, 16] extract hand-crafted protein features and apply machine learning models such as K-Nearest Neighbors (KNN) [17], Random Forest (RF) [18], Logistic Regression (LR) [19], Support Vector Machine (SVM) [20], Naive Bayesian (NB) [21], and Artificial Neural Networks (ANN) [22]. Recently, deep learning has shown promising performance in related tasks such as drug–target interaction prediction [23, 24], miRNA-disease association prediction [25, 26], and drug–drug interaction prediction [27, 28]. Among various deep learning architectures, convolutional neural networks (CNNs) have been used for potential biological interaction prediction due to their ability to capture rich local features through convolution operations. For example, PredPHI [29] and PHIAF [30] obtain hand-crafted genome and protein features from phages and bacteria, and use CNNs to build prediction models, while CL4PHI [31] employs frequency chaotic game representations as features. Moreover, graph neural networks have been applied to PBIs by constructing knowledge graphs from phage and bacterium sequences, enabling efficient propagation of biological features between nodes. For example, HostG [32], CHERRY [33], and PGCN [34] focus on constructing knowledge graphs, while PHPGCA [35], GERMAN-PHI [36], and PHISGAE [37] aim to address the sparsity of knowledge graphs.

However, the existing computational methods still have limitations. More specifically, certain methods rely on extracting hand-crafted features from phage and bacterial sequences, but these features often fail to reveal the complex biological patterns underlying the sequences. Moreover, CNN-based methods cannot effectively capture global information about PBIs. Furthermore, since most phages infect specific strains within a bacterial species [38], species-level prediction methods tend to assign multiple candidate phages to a given bacterial strain, and cannot find concrete infection.

In deep learning, the unified representation (UniRep) model [39] has been shown to effectively learn deep representations of protein sequences while preserving their physico-chemical and structural properties. Moreover, CNNs excel at extracting local feature maps from sequences through convolution operations [40], while the bi-directional gated recurrent unit (Bi-GRU) effectively captures long-term dependencies through a gating mechanism [41]. Furthermore, to address data imbalance, the synthetic minority oversampling technique (SMOTE) [42] is widely employed to generate minority class samples. Additionally, the attention mechanism enables deep neural networks to focus on task-relevant features, thereby improving performance.

In this article, inspired by the capabilities of UniRep, SMOTE, CNN, Bi-GRU, and the attention mechanism, we propose phage–bacterium interactions predictor (PBIP). Specifically, PBIP employs a protein representation model to transform phage and bacterial protein sequences into deep embeddings. The CNN and Bi-GRU modules are applied to capture local and global sequence features, while the attention mechanism emphasizes informative features. In addition, SMOTE is adopted to address data imbalance. The main contributions are summarized as follows:

- We propose PBIP, a strain-level PBIs predictor that integrates deep protein sequence embeddings, enriched local and global sequence features, and the attention mechanism to enhance the prediction accuracy.
- In PBIP, we first identify the strain-level interactions through biological infection experiments and sequencing on Klebsiella pneumoniae isolated from the clinical environment of Xiangya Hospital (see our work [43]). Then, we use the pretrained UniRep model to extract protein sequence features with biological significance. Subsequently, we apply SMOTE to augment positive interactions and data imbalance in the embedding space. After that, we design a deep learning model that integrates a CNN module for local feature extraction, a Bi-GRU module for capturing long-term dependencies in both forward and backward directions, and an attention mechanism to emphasize important features. Finally, we employ a fully connected layer with a Sigmoid activation function for PBI prediction.
- Experiments on the strain-level and species-level datasets show that PBIP outperforms the state-of-the-art methods. Moreover, the case studies on test set imbalance ratios and sequence similarity validate the robustness and generalizability of PBIP, while the ablation studies show the effectiveness of its various components for PBI prediction.

The remainder of this article is organized as follows: Section “Materials and methods” presents the problem formulation, datasets, technical details of PBIP, and performance metrics. Section “Results and discussion” describes the baseline settings, experimental results, and discussions. Finally, Section “Conclusion” concludes the study and outlines potential future research directions.

## Materials and methods

In this section, we first introduce the representation of protein sequences and the PBI prediction problem, and then describe the datasets used in this study. Then, we present a novel deep learning framework, namely PBIP, for strain-level PBI prediction. Finally, we provide the performance evaluation metrics.

### Protein sequence representation and problem formulation

#### Protein sequence representation

In phage–bacterium systems, interactions are mainly mediated by receptor-binding proteins (RBPs) located on the phage surface, which specifically recognize and bind to designated receptors on the bacterial cell envelope [29, 44, 45]. Consequently, the protein sequences encoded by both phages and their bacterial hosts contain rich interaction-related signals, making them a valuable foundation for computational modeling.

In this study, a protein sequence is defined as $S = (a_{1}, a_{2}, \dots , a_{T})$, where each element $a_{t}$ ($1 \leq t \leq T$) corresponds to 1 of the 20 standard amino acids {A, R, N, D, C, E, Q, G, H, I, L, K, M, F, P, S, T, W, Y, V}, and $T$ denotes the length of the sequence.

#### Phage–bacterium interaction prediction

PBIs refer to the biological processes of phages infecting bacterial hosts, including stages such as adsorption, injection, replication, and release [46].

We formulate the PBI prediction as a binary classification task. More specifically, given a phage–bacterium pair $[P, B]$, where $P = (S_{1}^{P}, S_{2}^{P}, \dots , S_{N_{p}}^{P})$, and $B = (S_{1}^{B}, S_{2}^{B}, \dots , S_{N_{b}}^{B})$ represent the sets of protein sequences for the phage and bacterium, respectively, and $N_{p}$ and $N_{b}$ denote the number of proteins in the corresponding organisms, the PBI task is to predict the interaction label $y \in \{0, 1\}$. Here, $y = 1$ indicates a positive interaction (i.e. the phage infects the bacterium), while $y = 0$ indicates a negative interaction (i.e. no infection occurs).

### Dataset

#### Strain-level interaction dataset

We construct a strain-level interaction dataset based on experimental results obtained from high-throughput and automated phage–bacterium characterization systems, with details of the datasets and experiments available in our work [43]. Specifically, we isolate 125 Klebsiella pneumoniae strains from clinical samples collected at Xiangya Hospital, and enrich 104 phages from hospital sewage through centrifugation and membrane filtration. We confirm the infectivity of each phage by measuring plaque-forming units, i.e. the number of clear zones formed on agar plates due to bacterial lysis. Each interaction is tested using a double-layer agar assay, and images of the resulting plaques are analyzed with an automated plaque identification algorithm, which calculates plaque characteristics such as radius, area, and transmissivity. The algorithm generates a composite score, and interactions with a score >1.5 are classified as positive. Each phage–bacterium pair is tested in three independent experiments, and interactions with at least two positive replicates are defined as positive, whereas all others are considered negative.

After integrating the results of independent replicates, the final interaction matrix contains 125 bacterial strains and 104 phages, yielding 13 000 phage–bacterium pairs. Among them, 938 (accounting for 7.22% of all entries) are positive interactions, reflecting a high degree of resistance within the bacterial population. On average, each bacterial strain is susceptible to 9.02 phages, and each phage can infect $\sim $7.50 bacterial strains.

**Figure 1:**
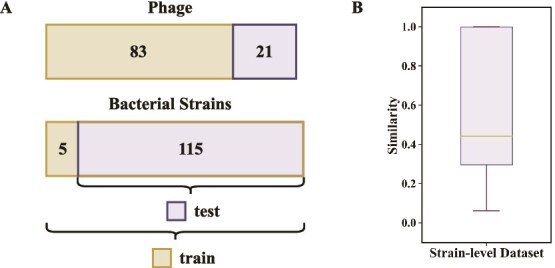
The strain-level interaction dataset, showing (A) the phages and bacteria in the training and test sets, respectively, and (B) the similarity between phages in the training and test sets.

We perform whole-genome sequencing and assembly for all bacterial strains and phages, followed by gene annotation using GeneMarkS [47] to obtain coding protein sequences. To ensure reliable interaction analysis, we remove five bacterial strains that exhibit complete resistance to all tested phages. The resulting dataset contains 104 phages and 120 bacterial strains, corresponding to 12 480 valid phage–bacterium records. Moreover, to minimize the risk of data leakage, we discard negative interactions involving phages with substantial overlap with the phages in the test set, and use the remaining interactions for model training. An overview of the dataset is provided in Table 1, and Fig. 1A illustrates the numbers of phages and bacteria in the training and test sets, distinguished by different colors.

**Table 1:** Detailed information of the strain-level training and test sets

Dataset	Number of positive interactions	Number of negative interactions	Total number of interactions
Training set	747	9213	9960
Test set	191	191	382

We evaluate the overall similarity between the training and test sets following prior studies [33, 35]. Specifically, we use Dashing [48] to compute sequence similarities, and for each phage in the test set, we record its highest similarity score with any phage in the train set. The distribution of these scores is shown in Fig. 1B. Only a small proportion of test phages display substantial similarity to training phages, with an average similarity of 0.60 and a median of 0.44. Generally, the dataset split under these settings on the strain-level interaction dataset is appropriate for training and evaluation.

#### Species-level interaction dataset

**Figure 2:**
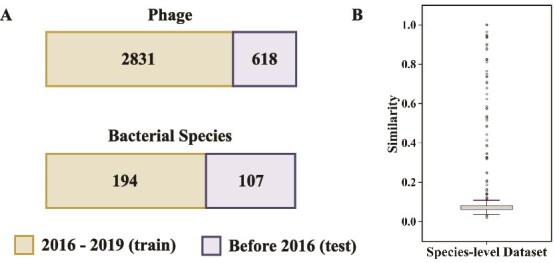
The species-level interaction dataset, showing (A) the phages and bacteria in the training and test sets, respectively, and (B) the similarity between phages in the training and test sets.

To evaluate the performance of PBIP and baseline methods in predicting PBIs at the species level, we adopt the benchmark dataset used in PredPHI [29] with 3449 phages and 301 bacterial species collected from the NCBI RefSeq database, and split data into training and test sets according to their submission date. More specifically, following PredPHI, we use the interactions between phages and bacteria submitted before 2016 as the independent test set, and adopt those submitted in or after 2016 as the train set. This split yields 2851 positive interactions for training and 618 for testing. In both sets, negative interactions are randomly selected from all unverified negative interactions, and the number of negative samples is matched to that of positive samples to address data imbalance. An overview of the dataset is presented in Table 2, while Fig. 2A shows the distribution of phages and bacteria in the train and test sets.

**Table 2:** Detailed information of the species-level training and test sets

Dataset	Number of positive interactions	Number of negative interactions	Total number of samples
Training set	2851	2851	5702
Test set	618	618	1236

Similar to the strain-level dataset, sequence similarity between phages in the training and test sets is assessed using Dashing [48]. The similarity distribution is shown in Fig. 2B. Only a small fraction of test phages show substantial similarity to those in the training set, with an average similarity of 0.12 and a median of 0.06, confirming that the dataset split is suitable for model training and evaluation.

### Phage–bacterium interactions framework

**Figure 3:**
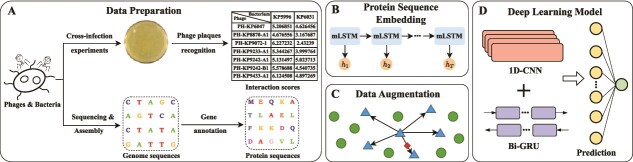
Overview of PBIP framework for predicting strain-level PBIs. (A) Collecting used strain-level data. (B) Generating protein sequence embeddings using deep representation learning. (C) Utilizing SMOTE to alleviate data imbalance in the embedding space. (D) Developing a deep learning model for predicting PBIs.

The model architecture of PBIP, as shown in Fig. 3, consists of four key components: (i) data preparation (see Fig. 3A), (ii) protein sequence embedding (see Fig. 3B), (iii) data augmentation (see Fig. 3C), and (iv) deep learning model (see Fig. 3D). More specifically, PBIP first obtains strain-level interactions as described in Section “Dataset”. Then, PBIP utilizes the pretrained UniRep model to generate deep embeddings of proteins. Next, to address the data imbalance issue, PBIP applies SMOTE to augment the positive interactions in the embedding space. Subsequently, the protein sequence embeddings are fed into a CNN module to capture local feature maps and a Bi-GRU module to extract long-term dependencies in both forward and backward directions. The resulting feature vectors are then processed by an attention module to emphasize informative features. Finally, the output vectors are fed into a fully connected layer with a sigmoid activation function to integrate phage and bacterial information for interaction prediction.

### Protein sequence embedding

**Figure 4:**
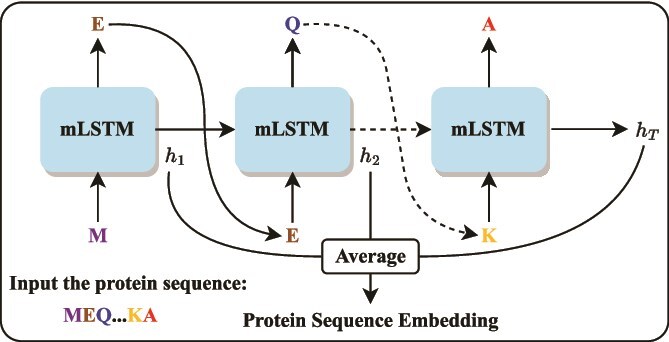
Overview of generating a protein sequence embedding using UniRep.

Many methods for predicting PBIs rely on hand-crafted features, which often fail to capture complex biological patterns in protein sequences. To overcome this limitation, we employ UniRep [39], a pretrained protein representation model that encodes sequences into 1900-dimensional embeddings while preserving physico-chemical and structural properties. UniRep leverages a multiplicative LSTM (mLSTM) [49] trained to predict the next amino acid, thereby learning contextualized representations from raw sequences. The embedding process is illustrated in Fig. 4.

Formally, given a protein sequence of a phage or bacterium $S=(a_{1},a_{2}, \dots , a_{T})$ with length $T$, where each element $a_{t}$ ($1 \leq t \leq T$) represents 1 of the 20 amino acids, we compute the embedding representation as follows. First, the protein sequence $S$ is one-hot encoded into a matrix $X_{\mathrm{one}} \in \mathbb{R}^{T\times 20}$ as follows: 


(1) \begin{align*}& X_{\mathrm{one}}=\mathrm{OneHot}(S) = [E_{1}; E_{2}; \dots; E_{T}],\end{align*}

where $E_{t}$ is a binary vector of length 20 representing the amino acid $a_{t}$ in the sequence $S$. The element $E_{tj}$, $1 \leq j \leq 20$, in the $j$th position of $E_{t}$ is set to 1 if it corresponds to $a_{t}$, and 0 otherwise.

Next, UniRep employs an embedding layer to convert the one-hot encoding matrix $X_{\mathrm{one}}$ into a continuous representation matrix $X_{\mathrm{emb}} \in \mathbb{R}^{T\times 10}$. This embedding layer is parameterized by a weight matrix $W_{\mathrm{emb}} \in \mathbb{R}^{20\times 10}$, where each row corresponds to the continuous representation of an amino acid. In UniRep, $W_{\mathrm{emb}}$ is initialized with randomly generated values and is updated during training. Specifically, each one-hot vector $E_{t}$ in $X_{\mathrm{one}}$ is replaced with its corresponding continuous vector from $W_{\mathrm{emb}}$, as shown below: 


(2) \begin{align*}& X_{\mathrm{emb}}=X_{\mathrm{one}} W_{\mathrm{emb}} = [X_{1}; X_{2}; \dots; X_{T}],\end{align*}

where $X_{t}$ represents the continuous embedding of the amino acid $a_{t}$.

Subsequently, UniRep employs an mLSTM with 1900 hidden units to process the sequence embeddings and generate contextualized states $h_{t}$, and the final protein embedding $x$ is obtained by averaging them: 


(3) \begin{align*}& \begin{aligned} h_{t} &= \mathrm{mLSTM}(X_{t}), \\ x &= \tfrac{1}{T}\sum_{t=1}^{T} h_{t}. \end{aligned}\end{align*}

Details of the processes of mLSTM in UniRep can be found in the Supplementary data.

We employ the officially released pretrained UniRep weights (https://github.com/churchlab/UniRep), which include both $W_{\mathrm{emb}}$ and the mLSTM parameters. Using these pretrained weights ensures the preservation of biologically meaningful features.

Since each phage or bacterium contains multiple proteins, we compute organism-level embeddings by averaging the corresponding protein embeddings. Specifically, we let $x_{pi}$ and $x_{bi}$ denote the UniRep embeddings of the $i$th protein in a phage and bacterium, respectively. The organism-level protein embeddings are calculated as follows: 


(4) \begin{align*}& \begin{aligned} x_{p} = \frac{1}{N_{p}} \sum^{N_{p}}_{i=1} x_{pi}, \\ x_{b} = \frac{1}{N_{b}} \sum^{N_{b}}_{i=1} x_{bi}, \end{aligned}\end{align*}

where $N_{p}$ and $N_{b}$ denote the number of protein sequences in the phage and bacterium, respectively. The combined embedding for a phage–bacterium pair $[P_{k}, B_{k}]$ is represented as $[x_{p}, x_{b}]_{k}$.

### Data augmentation

**Figure 5:**
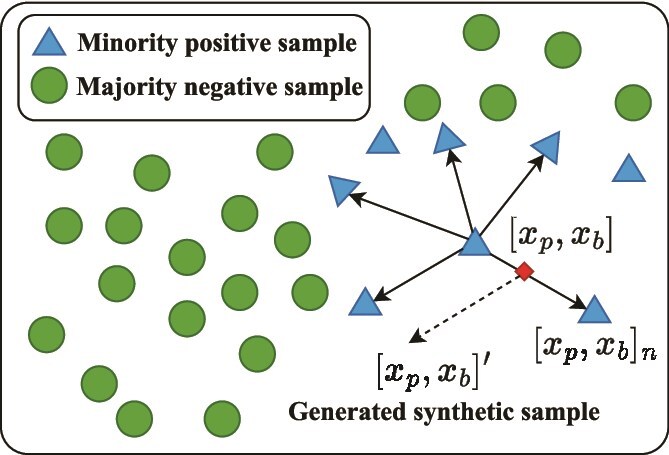
Overview of generating a synthetic sample using SMOTE in the embedding space.

The strain-level dataset exhibits a significant imbalance between positive and negative phage–bacterium pairs. To mitigate this issue, many techniques for tackling imbalance can be used. Here, SMOTE [42] is adopted due to its simplicity and effectiveness for tackling related bioinformatics prediction tasks, such as phage virion proteins prediction [50] and cell wall lytic enzymes prediction [51]. More specifically, we apply SMOTE [42] to generate synthetic positive interactions and balance the training set in the embedding space, while retaining biologically validated negative interactions. Importantly, the test set remains unchanged and contains only experimentally validated positive interactions. The SMOTE workflow is illustrated in Fig. 5.

Given an original positive sample $[x_{p}, x_{b}]$ and one of its nearest neighbors ${[x_{p}, x_{b}]}_{n}$, SMOTE creates a synthetic sample in the embedding space by linear interpolation: 


(5) \begin{align*}& [x_{p}, x_{b}]^{\prime} = [x_{p}, x_{b}] + \alpha \cdot \left( {[x_{p}, x_{b}]}_{n} - [x_{p}, x_{b}] \right),\end{align*}

where $\alpha \in [0,1]$ is a random coefficient. The number of synthetic samples is selected to ensure that the number of positive and negative samples is equal in the training set.

### Deep learning model

**Figure 6:**
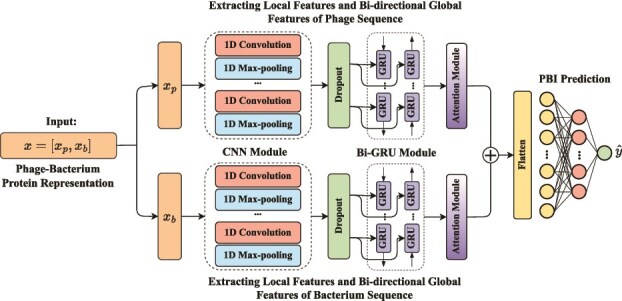
Overview of the proposed deep learning model architecture.

In this section, we present the proposed deep learning model for predicting PBIs. The model takes as input phage–bacterium protein embedding pairs $\{[x_{p}, x_{b}]_{k} \}_{k=1}^{N}$ derived from the pretrained UniRep. These embeddings are first processed through a CNN module to extract local features and then fed into a Bi-GRU module to capture dependencies in both forward and backward directions. Next, the resulting feature vectors are passed through an attention module to emphasize critical features. Finally, the output is fed into a fully connected layer with a sigmoid activation function to predict interaction probabilities. The overall architecture is illustrated in Fig. 6.

#### Convolutional neural network module

The CNN module consists of four 1D convolutional layers with max-pooling to extract local feature patterns. Each convolution layer computes feature maps by applying convolution filters to the input embeddings. The operation is defined as: 


(6) \begin{align*}& \begin{aligned} x^{C}_{t} =\mathrm{ReLU} (W^{C} \ast{x_{i}} + b^{C}), \\{x}^{C} = [x^{C}_{1}, x^{C}_{2}, \dots, x^{C}_{t}, \dots, x^{C}_{N_{f}}], \end{aligned}\end{align*}

where $x_{i}$ denotes either the phage protein embedding $x_{p}$ or the bacterium protein embedding $x_{b}$, $W^{C}$ and $b^{C}$ are the convolutional kernel weights and bias parameters, respectively, and $\ast $ denotes the convolution operation. The output $x^{C}_{t}$ represents the $t$th local feature, and $x^{C}$ denotes the resulting feature map composed of $N_{f}$ feature patterns.

A 1D max-pooling layer follows each convolutional layer to reduce the dimensionality of the feature map while preserving important features by selecting the maximum value over a sliding window. Moreover, a dropout layer is introduced after the CNN module to prevent overfitting by setting certain network weights to zero [52].

These local features learned by the CNN may capture biologically relevant short motifs within protein sequences [53], such as receptor-binding motifs in phage tail proteins. These patterns play key roles in phage adsorption and host recognition, and are informative for predicting infectivity.

#### Bi-directional gated recurrent unit module

**Figure 7:**
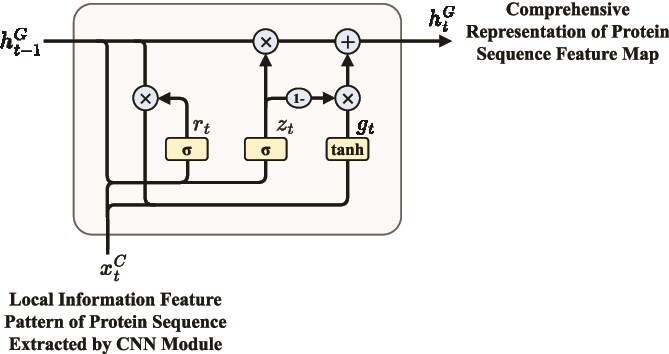
The architecture of the GRU cell.

Following the CNN module, a Bi-GRU is employed to capture long-term dependencies from both forward and backward directions. The internal structure of the GRU cell is presented in Fig. 7.

Formally, given the local feature map extracted by the CNN, denoted as ${x}^{C} = [x^{C}_{1}, x^{C}_{2}, \dots , x^{C}_{N_{f}}]$, the Bi-GRU processes the sequence bidirectionally. At each time step $t$, the GRU computes: 


(7) \begin{align*} r_{t} &= \sigma(W_{r} x^{C}_{t} + U_{r} h^{G}_{t-1} + b_{r}), \notag \\ z_{t} &= \sigma(W_{z} x^{C}_{t} + U_{z} h^{G}_{t-1} + b_{z}), \\ g_{t} &= \tanh(W_{g} x^{C}_{t} + U_{g} (r_{t} \odot h^{G}_{t-1}) + b_{g}), \notag \\ h^{G}_{t} &= (1 - z_{t}) \odot h^{G}_{t-1} + z_{t} \odot g_{t}, \notag\end{align*}

where $r_{t}$, $z_{t}$, $g_{t}$, and $h^{G}_{t}$ represent the reset gate, update gate, memory content, and hidden state at time step $t$, respectively. $W_{r}$, $W_{z}$, and $W_{g}$ are the weights of $x^{C}_{t}$, $U_{r}$, and $U_{z}$ are the weights of the $h^{G}_{t-1}$, while $U_{g}$ is the weight of the $r_{t} \odot h^{G}_{t-1}$. $b_{r}$, $b_{z}$, and $b_{g}$ are the corresponding biases for the reset gate, update gate, and memory content, respectively. $\odot $ denotes element-wise multiplication, $\sigma (\cdot )$ and $\tanh (\cdot )$ represent the sigmoid and hyperbolic tangent functions, respectively. Details of the GRU computation process can be found in the Supplementary data.

Bi-GRU aggregates information from both directions by concatenating the forward and backward hidden states at each step: 


(8) \begin{align*}& h^{G}_{t} = \overrightarrow{h^{G}_{t}} \oplus \overleftarrow{h^{G}_{t}}.\end{align*}

where $\oplus $ denotes the concatenation operation.

The Bi-GRU captures long-term dependencies across the protein sequence [54], enabling the extraction of global features that complement local motifs learned by CNN. Such features help identify PBIs formed by distant but functionally coordinated residues within RBPs.

#### Attention module

To emphasize informative features essential for PBI prediction, an attention mechanism is applied after the Bi-GRU module. It computes a weighted sum of all hidden states, where higher weights are assigned to more relevant features: 


(9) \begin{align*}& h^{A} = \sum_{t = 1}^{N_{f}} \alpha^{A}_{t} h^{G}_{t},\end{align*}

where $\alpha ^{A}_{t}$ denotes the attention weight with $\sum _{t=1}^{N_{f}} \alpha ^{A}_{t} = 1$. The output vector $h^{A}$ reflects the relative importance of each feature in the sequence.

#### Phage–bacterium interaction prediction

The representations of phage and bacterium, $h^{A}_{p}$ and $h^{A}_{b}$, obtained through CNN, Bi-GRU, and attention modules, capture local features, long-term dependencies, and key interaction-relevant features. These are concatenated and fed into a fully connected layer to compute the interaction probability: 


(10) \begin{align*}& \hat y = \sigma( W_{f}(h^{A}_{p} \oplus h^{A}_{b}) + b_{f}),\end{align*}

where $W_{f}$ and $b_{f}$ are the weight and bias of the fully connected layer, respectively.

#### Model training

The binary cross-entropy loss function is employed to compute the error between the interaction scores predicted by PBIP and the true labels, defined as: 


(11) \begin{align*}& \mathrm{Loss} = - \frac{1}{N} \sum_{k=1}^{N} \left( y_{k} \cdot \log{\hat{y}}_{k} + (1 - y_{k}) \cdot \log (1 - {\hat{y}}_{k}) \right),\end{align*}

where $y_{k} \in \{ 0,1\}$ represents the true label of the $k$th phage–bacterium pair (0 for negative and 1 for positive interaction). $\hat{y}_{k}$ is the predicted probability of a positive interaction, and $N$ is the total number of samples.

The AMSGrad optimizer [55] is utilized to minimize the loss function. Model hyperparameters are summarized in Table 3. PBIP is implemented in Python 3.8.10 with Keras 2.8.0 and trained on an NVIDIA RTX 3090 GPU.

**Table 3:** Network module parameters and training hyperparameters

Network module			Parameters			Values
1D-CNN			Layer number			4
			Kernel size			3
			Pooling size			2
			Filter number			[32, 64, 128, 256]
			Dropout rate			0.5
Bi-GRU			Hidden size			64
			Dropout rate			0.5
Attention			Neurons			32
Training hyperparameters			Learning rate			3e-4
			Batch size			16
			Epoch			200

### Performance evaluation metrics

To evaluate the performance of PBIP and baseline methods, eight evaluation metrics are used, namely Accuracy, Precision, Sensitivity (Recall), Specificity, F1-score, Matthews correlation coefficient (MCC), Area under the receiver operating characteristic curve (AUC), and Area under the precision-recall curve (AUPR). These metrics are calculated as follows: 


(12) \begin{align*} & \mathrm{Accuracy}=\frac{\mathrm{TP}+\mathrm{TN}}{\mathrm{TP}+\mathrm{TN}+\mathrm{FP}+\mathrm{FN}}, \end{align*}

(13) \begin{align*} & \mathrm{Precision}=\frac{\mathrm{TP}}{\mathrm{TP}+\mathrm{FP}}, \end{align*}

(14) \begin{align*} & \mathrm{Sensitivity} \ (\mathrm{Recall})=\frac{\mathrm{TP}}{\mathrm{TP}+\mathrm{FN}}, \end{align*}

(15) \begin{align*} & \mathrm{Specificity}=\frac{\mathrm{TN}}{\mathrm{FP}+\mathrm{TN}}, \end{align*}

(16) \begin{align*} & \mathrm{F1-score}=\frac{2 \times \mathrm{Precision} \times \mathrm{Recall}}{\mathrm{Precision}+\mathrm{Recall}}, \end{align*}

(17) \begin{align*} & \mathrm{MCC}=\frac{\mathrm{TP} \times \mathrm{TN} -\mathrm{FP} \times \mathrm{FN}}{\sqrt{(\mathrm{TP}+\mathrm{FP})(\mathrm{TP}+\mathrm{FN})(\mathrm{TN}+\mathrm{FP})(\mathrm{TN}+\mathrm{FN})}}, \end{align*}

where TP, TN, FP, and FN denote true positives, true negatives, false positives, and false negatives, respectively. In addition to the above metrics, the ROC curve plots true positive rate against false positive rate, with AUC representing the area under this curve. The precision-recall curve plots precision versus recall, and AUPR is its corresponding area.

## Results and discussion

In this section, we validate the effectiveness of the proposed PBIP by making a comparison with the state-of-the-art PBI prediction methods. Moreover, certain comparative experiments are conducted to show the impact of test set imbalance as well as training and test similarity on prediction performance. Finally, an ablation study is performed to assess the necessity of each component of PBIP in improving the prediction performance. In the following, the baseline methods are first introduced, and then the results and discussion of all methods are given.

### Baseline settings

To validate the effectiveness of PBIP, we compare it with four state-of-the-art PBI prediction methods: LeiteANN [15], LeiteBagging [16], PredPHI [29], and PHIAF [30]. Given that all negative interactions in the strain-level dataset are experimentally validated, we further construct SMOTE-based variants of PredPHI and PHIAF, named PredPHIS and PHIAFS, which generate synthetic positive interactions in the embedding space to achieve data balance. Then, to further validate the performance of PBIP, we compare it with five widely used machine learning classifiers, namely LR [19], SVM [56], KNN [17], RF [18], and eXtreme Gradient Boosting (XGB) [57].

LeiteANN [15]: This method extracts three hand-crafted features including amino acid composition (AAC), chemical composition (AC), and molecular weight (MW). The training set is balanced by randomly selecting negative interactions, and an ANN model is trained for PBI prediction.

LeiteBagging [16]: It adopts the same feature set and negative sampling strategy as LeiteANN. Instead of a single classifier, it employs a Bagging ensemble with hard voting that combines RF, ANN, and KNN.

PredPHI [29]: This method uses AAC, AC, and MW features, balances the training set through negative sampling, and trains a CNN for prediction.

PHIAF [30]: In addition to the features used in PredPHI, it also extracts seven DNA-based features including $k$-mer, reverse complement $k$-mer (RCKmer), nucleic acid composition, di-nucleotide composition, tri-nucleotide composition, composition of $p$-spaced nucleic acid pairs, and electron–ion interaction pseudopotentials of trinucleotides (PseEIIP). The training set is balanced using negative sampling, and prediction is performed with a CNN incorporating an attention mechanism.

PredPHIS: This variant of PredPHI generates positive interactions in the embedding space using SMOTE to balance the training set. The feature extraction and deep learning model are identical to those employed in PredPHI.

PHIAFS: This variant of PHIAF applies SMOTE to generate positive interactions, with feature extraction and model components identical to those used in PHIAF.

LR [19], SVM [56], KNN [17], RF [18], and XGB [57]: These methods use UniRep-based protein embeddings, balance the training set through negative sampling, and are trained as individual models for PBI prediction.

To ensure fair comparison, all baselines are retrained on the same strain-level dataset, with consistent train/test partitioning as in our method. For the species-level dataset, since the numbers of positive and negative interactions are balanced, SMOTE-based data augmentation is removed.

### Performance comparison on cross-validation

To ensure stable training and assess the performance of PBIP, we perform 10-fold cross-validation on both the strain-level and species-level datasets. The data are randomly partitioned into 10 subsets. In each round, the model uses nine subsets for training and the remaining one for validation. The mean results over all folds are reported for each dataset. As shown in Table 4, PBIP achieves the best results on most evaluation metrics across both datasets. Specifically, on the strain-level dataset, PBIP attains the highest Accuracy (0.96), Sensitivity (0.90), F1-score (0.86), MCC (0.72), and AUC (0.96). On the species-level dataset, it obtains the best Accuracy (0.92), Precision (0.92), Sensitivity (0.92), F1-score (0.92), MCC (0.85), AUC (0.96), and AUPR (0.98).

**Table 4:** Performance comparison between PBIP and baseline methods on strain-level and species-level training sets using 10-fold cross-validation

Level	Model	Accuracy	Precision	Sensitivity	Specificity	F1-score	MCC	AUC	AUPR
strain	LeiteANN	0.70$\pm $0.05	0.70$\pm $0.05	0.70$\pm $0.05	0.67$\pm $0.07	0.69$\pm $0.05	0.39$\pm $0.10	0.75$\pm $0.05	0.76$\pm $0.06
	LeiteBagging	0.83$\pm $0.02	**0.83$\pm $0.02**	0.83$\pm $0.02	0.82$\pm $0.04	0.83$\pm $0.02	0.66$\pm $0.05	–	–
	PredPHI	0.83$\pm $0.02	**0.83$\pm $0.02**	0.83$\pm $0.02	0.83$\pm $0.03	0.83$\pm $0.02	0.66$\pm $0.04	0.90$\pm $0.03	**0.91$\pm $0.03**
	PHIAF	0.80$\pm $0.03	0.80$\pm $0.06	0.79$\pm $0.06	0.80$\pm $0.07	0.80$\pm $0.03	0.60$\pm $0.07	0.88$\pm $0.04	0.88$\pm $0.03
	PredPHIS	0.92$\pm $0.01	0.73$\pm $0.03	0.87$\pm $0.02	0.93$\pm $0.01	0.78$\pm $0.03	0.59$\pm $0.05	0.94$\pm $0.01	0.69$\pm $0.07
	PHIAFS	0.95$\pm $0.01	0.63$\pm $0.07	0.69$\pm $0.07	**0.97$\pm $0.01**	0.66$\pm $0.03	0.63$\pm $0.04	0.92$\pm $0.02	0.72$\pm $0.07
	LR	0.69$\pm $0.02	0.69$\pm $0.03	0.69$\pm $0.03	0.65$\pm $0.03	0.68$\pm $0.03	0.37$\pm $0.05	0.74$\pm $0.04	0.72$\pm $0.05
	SVM	0.77$\pm $0.04	0.78$\pm $0.04	0.77$\pm $0.04	0.82$\pm $0.04	0.77$\pm $0.04	0.55$\pm $0.09	0.84$\pm $0.03	0.85$\pm $0.03
	KNN	0.76$\pm $0.02	0.77$\pm $0.02	0.76$\pm $0.02	0.67$\pm $0.05	0.76$\pm $0.02	0.53$\pm $0.04	0.87$\pm $0.01	0.86$\pm $0.03
	RF	0.79$\pm $0.03	0.79$\pm $0.03	0.79$\pm $0.03	0.78$\pm $0.05	0.79$\pm $0.03	0.59$\pm $0.06	0.86$\pm $0.02	0.83$\pm $0.03
	XGB	0.79$\pm $0.03	0.79$\pm $0.03	0.79$\pm $0.04	0.79$\pm $0.04	0.79$\pm $0.03	0.59$\pm $0.07	0.87$\pm $0.03	0.87$\pm $0.02
	PBIP	**0.96$\pm $0.01**	0.83$\pm $0.03	**0.90$\pm $0.02**	0.96$\pm $0.01	**0.86$\pm $0.02**	**0.72$\pm $0.04**	**0.96$\pm $0.01**	0.83$\pm $0.04
species	LeiteANN	0.84$\pm $0.01	0.85$\pm $0.01	0.84$\pm $0.02	0.81$\pm $0.02	0.84$\pm $0.01	0.69$\pm $0.03	0.93$\pm $0.01	0.92$\pm $0.02
	LeiteBagging	0.89$\pm $0.01	0.90$\pm $0.01	0.89$\pm $0.02	0.82$\pm $0.03	0.89$\pm $0.02	0.79$\pm $0.03	–	–
	PredPHI	0.91$\pm $0.02	0.91$\pm $0.02	0.91$\pm $0.02	0.90$\pm $0.04	0.91$\pm $0.02	0.82$\pm $0.04	0.97$\pm $0.01	0.97$\pm $0.01
	PHIAF	0.91$\pm $0.01	0.92$\pm $0.02	0.90$\pm $0.04	0.92$\pm $0.02	0.91$\pm $0.02	0.82$\pm $0.02	**0.98$\pm $0.01**	**0.98$\pm $0.01**
	LR	0.85$\pm $0.02	0.85$\pm $0.02	0.85$\pm $0.02	0.85$\pm $0.02	0.85$\pm $0.02	0.70$\pm $0.03	0.92$\pm $0.01	0.89$\pm $0.03
	SVM	0.86$\pm $0.01	0.86$\pm $0.01	0.86$\pm $0.01	0.88$\pm $0.02	0.86$\pm $0.01	0.73$\pm $0.03	0.95$\pm $0.01	0.95$\pm $0.01
	KNN	0.86$\pm $0.01	0.86$\pm $0.01	0.86$\pm $0.01	0.79$\pm $0.01	0.86$\pm $0.01	0.72$\pm $0.02	0.93$\pm $0.01	0.90$\pm $0.02
	RF	0.88$\pm $0.01	0.89$\pm $0.01	0.88$\pm $0.01	**0.95$\pm $0.01**	0.88$\pm $0.01	0.77$\pm $0.03	0.93$\pm $0.01	0.90$\pm $0.02
	XGB	0.89$\pm $0.01	0.89$\pm $0.01	0.89$\pm $0.01	0.92$\pm $0.02	0.89$\pm $0.01	0.79$\pm $0.03	0.97$\pm $0.01	0.96$\pm $0.01
	PBIP	**0.92$\pm $0.01**	**0.92$\pm $0.01**	**0.92$\pm $0.01**	0.92$\pm $0.01	**0.92$\pm $0.01**	**0.85$\pm $0.03**	**0.98$\pm $0.01**	**0.98$\pm $0.01**

The best performance is highlighted in boldface. “–” represents that the metric is not available.

**Table 5:** Performance comparison between PBIP and baseline methods on strain-level and species-level test sets

Level	Model	Accuracy	Precision	Sensitivity	Specificity	F1-score	MCC	AUC	AUPR
strain	LeiteANN	0.64	0.64	0.64	0.63	0.64	0.29	0.68	0.73
	LeiteBagging	0.72	0.73	0.72	0.85	0.71	0.45	–	–
	PredPHI	0.76	0.77	0.76	0.85	0.76	0.53	0.81	0.84
	PHIAF	0.73	0.82	0.59	0.87	0.69	0.48	0.80	0.81
	PredPHIS	0.77	0.80	0.77	0.93	0.76	0.57	**0.89**	**0.89**
	PHIAFS	0.75	**0.91**	0.55	**0.95**	0.69	0.54	0.85	0.86
	LR	0.63	0.63	0.63	0.60	0.63	0.26	0.66	0.60
	SVM	0.72	0.73	0.72	0.80	0.72	0.44	0.80	0.83
	KNN	0.72	0.72	0.72	0.74	0.72	0.44	0.78	0.76
	RF	0.73	0.75	0.73	0.88	0.73	0.49	0.82	0.83
	XGB	0.75	0.76	0.75	0.83	0.75	0.50	0.83	0.84
	PBIP	**0.80**	0.81	**0.80**	0.89	**0.80**	**0.61**	0.86	**0.89**
species	LeiteANN	0.65	0.67	0.65	0.79	0.65	0.32	0.74	0.73
	LeiteBagging	0.66	0.67	0.66	0.78	0.66	0.34	–	–
	PredPHI	0.66	0.67	0.66	0.77	0.66	0.33	0.76	0.71
	PHIAF	0.68	0.67	**0.71**	0.64	**0.69**	0.36	0.75	0.72
	LR	0.52	0.52	0.52	0.53	0.52	0.03	0.52	0.48
	SVM	0.60	0.65	0.60	0.88	0.57	0.25	0.70	0.67
	KNN	0.59	0.66	0.59	0.92	0.54	0.24	0.71	0.66
	RF	0.58	**0.70**	0.58	**0.97**	0.51	0.25	0.74	0.72
	XGB	0.62	0.66	0.62	0.88	0.60	0.28	0.69	0.69
	PBIP	**0.69**	**0.70**	0.69	0.77	**0.69**	**0.39**	**0.78**	**0.75**

The best performance is highlighted in boldface. “–” represents that the metric is not available.

The results in Table 4 further demonstrate that deep learning-based approaches (PBIP, PredPHI, PHIAF, PredPHIS, and PHIAFS) generally outperform machine learning-based methods (LeiteANN, LeiteBagging, LR, SVM, KNN, RF, and XGB). Among the machine learning baselines, LeiteBagging and XGB perform particularly well. This is likely due to the advantages of ensemble learning in enhancing generalization (LeiteBagging) and the integration of UniRep protein embeddings with L1/L2 regularization in XGB. Moreover, among the methods that employ SMOTE to balance the datasets, PBIP outperforms both PredPHIS and PHIAFS. These findings highlight that the combination of UniRep protein embeddings, CNN, Bi-GRU, and the attention mechanism is more effective for predicting PBIs.

### Performance comparison on the test set

To further evaluate the performance of each method, we test them on two independent test sets, which are carefully partitioned to ensure a robust assessment. As shown in Table 5, PBIP achieves the best performance across most evaluation metrics on both the strain-level and species-level datasets. Specifically, it achieves the highest Accuracy (0.80), Sensitivity (0.80), F1-score (0.80), MCC (0.61), and AUPR (0.89) on the strain-level dataset, as well as the highest Accuracy (0.69), Precision (0.70), F1-score (0.69), MCC (0.39), AUC (0.78), and AUPR (0.75) on the species-level dataset. Notably, on the strain-level dataset, the SMOTE-based variants PredPHIS and PHIAFS outperform their original counterparts PredPHI and PHIAF across most metrics, underscoring the positive role of data balancing strategies in improving prediction performance.

**Figure 8:**
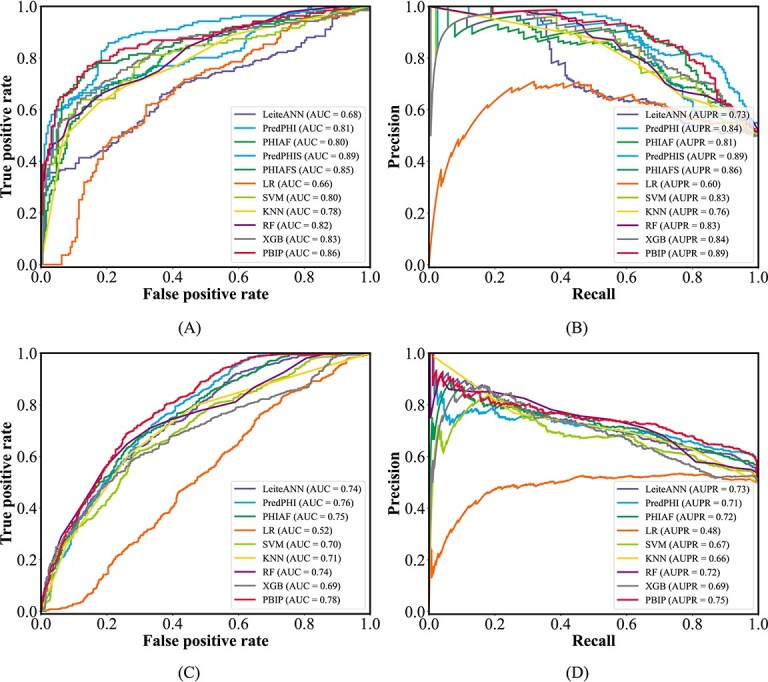
Performance comparison between PBIP and baseline methods based on ROC and PR curves on strain-level and species-level test sets: (A) strain-level ROC, (B) strain-level PR, (C) species-level ROC, and (D) species-level PR.

To visually compare various methods, we present the ROC and PR curves on the strain-level and species-level test sets in Fig. 8. As shown in Fig. 8, PBIP achieves the highest AUPR (0.89) on the strain-level set and the highest AUC (0.78) and AUPR (0.75) on the species-level set. These results further validate the effectiveness of the proposed deep learning framework in predicting PBIs.

### Impact of test set imbalance on prediction performance

In real-world scenarios, the number of positive interactions is substantially lower than that of negative interactions. To evaluate the performance of PBIP and baseline methods under imbalanced test conditions, we conduct a case study using the strain-level interaction dataset. Specifically, we incorporate negative interactions discarded from the training set to create nine imbalanced test sets, with positive-to-negative interaction ratios ranging from 1:2 to 1:10. Moreover, we use MCC to evaluate model performance on imbalanced datasets.

**Figure 9:**
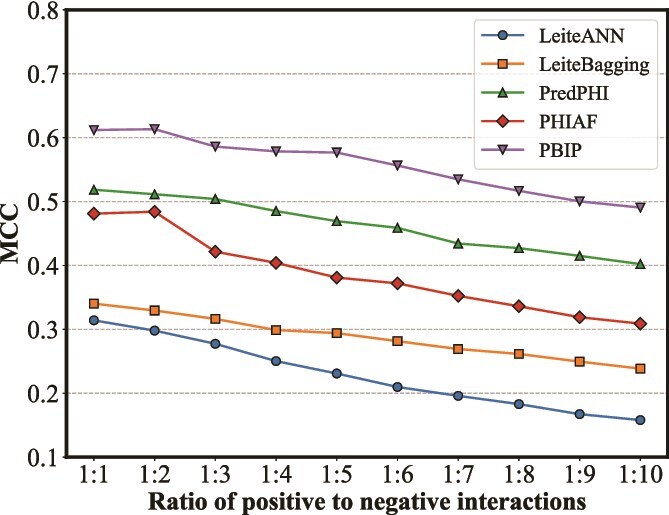
Performance comparison between PBIP and baseline methods at different test set imbalance ratios on strain-level dataset.

As shown in Fig. 9, the MCC decreases consistently as the degree of imbalance increases. Notably, PBIP surpasses all state-of-the-art methods across all imbalanced test sets, demonstrating robust performance under varying imbalance ratios and highlighting its potential applicability in real-world scenarios where positive interactions are inherently scarce.

### Impact of training and test similarity on prediction performance

We utilize Dashing [48] to quantify the genomic similarity between training and test sets. For each phage in the test set, we calculate its similarity score with every phage in the training set and retain the highest value as its representative similarity. Based on these scores, the test phages are partitioned into five similarity intervals, and the predictive performance is evaluated separately at both strain and species levels.

**Figure 10:**
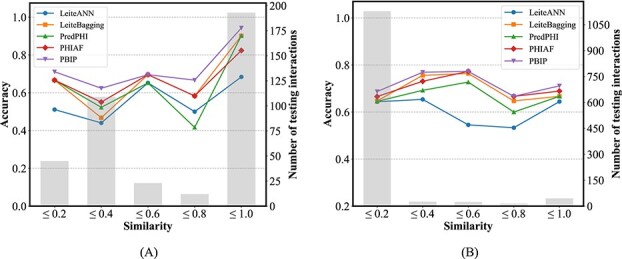
Performance comparison between PBIP and baseline methods at different similarity intervals on (A) strain-level and (B) species-level datasets.

As shown in Fig. 10, higher similarity between training and testing data generally corresponds to improved predictive accuracy across all methods. PBIP consistently achieves the highest accuracy within most similarity ranges, further confirming its advantage over competing approaches. However, an anomaly is observed in that performance does not strictly follow the expected upward trend with increasing similarity. This irregularity may arise from the relatively small number of test interactions within certain similarity intervals (as indicated by the gray bars), which can magnify the influence of sample-specific characteristics.

**Table 6:** Performance comparison between PBIP and the variant models on strain-level and species-level test sets

Level	Model	Accuracy	Precision	Sensitivity	Specificity	F1-score	MCC	AUC	AUPR
strain	PBIP1	0.77	0.78	0.77	0.83	0.77	0.55	0.82	0.85
	PBIP2	0.71	0.77	0.71	0.94	0.70	0.48	0.75	0.79
	PBIP3	0.71	0.77	0.71	**0.95**	0.69	0.48	0.83	0.85
	PBIP4	0.73	0.76	0.73	0.90	0.73	0.49	0.84	0.86
	PBIP5	0.74	0.76	0.74	0.87	0.74	0.50	0.81	0.80
	PBIP	**0.80**	**0.81**	**0.80**	0.89	**0.80**	**0.61**	**0.86**	**0.89**
species	PBIP1	0.60	0.60	0.60	0.68	0.60	0.21	0.65	0.62
	PBIP2	0.63	0.64	0.63	0.72	0.63	0.27	0.70	0.67
	PBIP3	0.64	0.64	0.64	0.66	0.63	0.27	0.71	0.66
	PBIP4	0.66	0.66	0.66	0.75	0.65	0.32	0.71	0.66
	PBIP	**0.69**	**0.70**	**0.69**	**0.77**	**0.69**	**0.39**	**0.78**	**0.75**

The best performance is highlighted in boldface

### Ablation study

The aforementioned experimental results demonstrate the effectiveness of PBIP in predicting PBIs. To further evaluate the contribution of each component, we conduct an ablation study. Notably, the CNN module is excluded from this study due to the considerable slowdown in training when feeding high-dimensional embedding representations directly into the Bi-GRU. The PBIP variants are as follows:

- PBIP1: This variant utilizes the same hand-crafted features as PredPHI.
- PBIP2: This variant employs the same hand-crafted features as PHIAF.
- PBIP3: This variant is without the Bi-GRU module.
- PBIP4: This variant is without the attention module.
- PBIP5: This variant is without the data augmentation module.

Table 6 presents the performance comparison between PBIP and its variant models on the test sets. Overall, PBIP consistently surpasses its variants across the majority of evaluation metrics. Specifically, the results of PBIP1 and PBIP2 highlight the superiority of UniRep-derived embeddings, which provide substantial performance gains over traditional hand-crafted features. Furthermore, the results of PBIP3, PBIP4, and PBIP5 demonstrate the individual contributions of the Bi-GRU module, the attention mechanism, and the data augmentation strategy. Notably, in the strain-level dataset, PBIP5 is trained without SMOTE, scaling down to the original validated positive interactions. Comparing PBIP5 to PBIP, the performance drops by $\sim $7.5%, demonstrating that SMOTE with data augmentation substantially enhances predictive performance.

## Conclusion

The PBI prediction task is of critical significance for advancing phage therapy. However, the existing computational methods mainly focus on species or higher-level classification resolutions, and often neglect the utilization of deep protein embedding representations. In this article, we have proposed PBIP, a novel deep learning framework designed for strain-level PBI prediction. PBIP first constructs a strain-level interaction dataset through biological infection experiments and sequencing of Klebsiella pneumoniae isolated from the clinical environment of Xiangya Hospital. Then, PBIP leverages the pretrained UniRep to generate deep embeddings of protein sequences, enabling the efficient capture of rich sequence-level biological patterns. To address data imbalance, SMOTE is employed to generate the additional positive interactions in the embedding space. Subsequently, these embedding feature vectors are fed into a CNN module to extract local features, a Bi-GRU module to capture long-term dependencies in both forward and backward directions, and an attention module to emphasize the contribution of key features. Finally, a fully connected layer with a sigmoid activation function tackles these vectors for PBI prediction. Extensive experimental results have demonstrated the superiority of PBIP over the state-of-the-art methods for PBI prediction. The case studies on test set imbalance ratios and sequence similarity have validated the robustness and generalizability of PBIP, while the ablation studies have demonstrated the effectiveness of its various components for PBI prediction.

Although PBIP achieves a competitive performance for PBI prediction, its interpretability for the presence of specific protein signatures for resisting certain phage types remains unresolved. In the future, we will systematically evaluate the predictive value of the genomic and domain-level features of proteins, aiming to enhance both prediction accuracy and biological interpretability.

**Key Points**

- We propose phage–bacterium interactions predictor (PBIP), a deep learning framework that leverages deep protein embeddings combined with the convolutional neural network, bi-directional gated recurrent unit, and attention modules to capture complex local and global features for accurate strain-level PBI prediction.
- To address data imbalance, PBIP applies the synthetic minority oversampling technique to augmenting positive interactions in the embedding space, enhancing model robustness and predictive performance.
- Extensive experiments on strain-level and species-level datasets show that PBIP outperforms the existing methods, with ablation studies validating the effectiveness of its deep embedding, deep neural network architecture, and data augmentation strategies.

## Supplementary Material

Supplementary_Document_bbaf656

## Data Availability

The code, validated positive and negative interactions at the strain level, experimental phenotypes, and species-level interaction data are available at https://github.com/a1678019300/PBIP. Moreover, all strain-level sequence data are publicly available from the China National GeneBank DataBase Sequence Archive (CNSA) [58] under accession number CNP0006217: https://db.cngb.org/search/project/CNP0006217/.

## References

[1] Kortright KE, Chan BK, Koff JL. et al. Phage therapy: a renewed approach to combat antibiotic-resistant bacteria. *Cell Host Microbe* 2019;25:219–32. 10.1016/j.chom.2019.01.014 30763536

[2] Pan J, You Z, You W. et al. PTBGRP: predicting phage–bacteria interactions with graph representation learning on microbial heterogeneous information network. *Brief Bioinform* 2023;24:bbad328. 37742053 10.1093/bib/bbad328

[3] Mallawaarachchi V, Roach MJ, Decewicz P. et al. Phables: from fragmented assemblies to high-quality bacteriophage genomes. *Bioinformatics* 2023;39:btad586. 37738590 10.1093/bioinformatics/btad586 PMC10563150

[4] Ma L, Deng W, Bai Y. et al. Identifying phage sequences from metagenomic data using deep neural network with word embedding and attention mechanism. *IEEE/ACM Trans Comput Biol Bioinform* 2023;20:3772–85. 10.1109/TCBB.2023.3322870 37812548

[5] Wang C, Zhang J, Cheng L. et al. DPProm: a two-layer predictor for identifying promoters and their types on phage genome using deep learning. *IEEE J Biomed Health Inform* 2022;26:5258–66. 10.1109/JBHI.2022.3193224 35867364

[6] Gaborieau B, Vaysset H, Tesson F. et al. Prediction of strain level phage–host interactions across the *Escherichia* genus using only genomic information. *Nat Microbiol* 2024;9:2847–61. 10.1038/s41564-024-01832-5 39482383

[7] Villarroel J, Kleinheinz KA, Jurtz VI. et al. Hostphinder: a phage host prediction tool. *Viruses* 2016;8:116. 27153081 10.3390/v8050116 PMC4885074

[8] Pons JC, Paez-Espino D, Riera G. et al. VPF-class: taxonomic assignment and host prediction of uncultivated viruses based on viral protein families. *Bioinformatics* 2021;37:1805–13. 10.1093/bioinformatics/btab026 33471063 PMC8830756

[9] Ahlgren NA, Ren J, Lu YY. et al. Alignment-free ${d}_2^{\ast }$ oligonucleotide frequency dissimilarity measure improves prediction of hosts from metagenomically-derived viral sequences. *Nucleic Acids Res* 2017;45:39–53. 27899557 10.1093/nar/gkw1002 PMC5224470

[10] Zielezinski A, Deorowicz S, Gudyś A. Phist: fast and accurate prediction of prokaryotic hosts from metagenomic viral sequences. *Bioinformatics* 2022;38:1447–9. 10.1093/bioinformatics/btab837 34904625 PMC8826084

[11] Paez-Espino D, Eloe-Fadrosh EA, Pavlopoulos GA. et al. Uncovering earth’s virome. Nature 2016;536:425–30. 10.1038/nature19094 27533034

[12] Shmakov SA, Sitnik V, Makarova KS. et al. The CRISPR spacer space is dominated by sequences from species-specific mobilomes. *MBio* 2017;8:10–1128. 10.1128/mBio.01397-17 PMC5605939 28928211

[13] Zhang R, Mirdita M, Karin EL. et al. SpacePHARER: sensitive identification of phages from CRISPR spacers in prokaryotic hosts. *Bioinformatics* 2021;37:3364–6. 10.1093/bioinformatics/btab222 33792634 PMC8504623

[14] Galiez C, Siebert M, Enault F. et al. WIsH: Who is the host? Predicting prokaryotic hosts from metagenomic phage contigs. *Bioinformatics* 2017;33:3113–4. 10.1093/bioinformatics/btx383 28957499 PMC5870724

[15] Leite DMC, Brochet X, Resch G. et al. Computational prediction of inter-species relationships through omics data analysis and machine learning. *BMC Bioinform* 2018;19:151–9. 10.1186/s12859-018-2388-7 PMC6245486 30453987

[16] Leite DMC, Lopez JF, Brochet X. et al. Exploration of multiclass and one-class learning methods for prediction of phage-bacteria interaction at strain level. In: 2018 IEEE International Conference on Bioinformatics and Biomedicine (BIBM), pp. 1818–25. Madrid, Spain: IEEE, 2018.

[17] Cover T, Hart P. Nearest neighbor pattern classification. *IEEE Trans Inform Theory* 1967;13:21–7. 10.1109/TIT.1967.1053964

[18] Breiman L . Random forests. *Mach Learn* 2001;45:5–32. 10.1023/A:1010933404324

[19] Hsiang-Fu Y, Huang F-L, Lin C-J. Dual coordinate descent methods for logistic regression and maximum entropy models. *Mach Learn* 2011;85:41–75. 10.1007/s10994-010-5221-8

[20] Hearst MA, Dumais ST, Osuna E. et al. Support vector machines. *IEEE Intell Syst Appl* 1998;13:18–28. 10.1109/5254.708428

[21] Rish I. et al. An empirical study of the naive Bayes classifier. In: IJCAI 2001 workshop on empirical methods in artificial intelligence, Vol. 3, pp. 41–6. Seattle, Washington, USA: Morgan Kaufmann, 2001.

[22] Witten IH, Frank E. Data mining: practical machine learning tools and techniques with java implementations. *ACM Sigmod Record* 2002;31:76–7.

[23] Yansen S, Zhiyang H, Wang F. et al. AMGDTI: drug–target interaction prediction based on adaptive meta-graph learning in heterogeneous network. *Brief Bioinform* 2024;25:bbad474. 10.1093/bib/bbad474 PMC10749791 38145949

[24] Zhang L, Wang C-C, Zhang Y. et al. GPCNDTA: prediction of drug-target binding affinity through cross-attention networks augmented with graph features and pharmacophores. *Comput Biol Med* 2023;166:107512. 10.1016/j.compbiomed.2023.107512 37788507

[25] Zhang G, Li M, Deng H. et al. SGNNMD: signed graph neural network for predicting deregulation types of miRNA-disease associations. *Brief Bioinform* 2022;23:bbab464. 34875683 10.1093/bib/bbab464

[26] Huang F, Yue X, Xiong Z. et al. Tensor decomposition with relational constraints for predicting multiple types of microRNA-disease associations. *Brief Bioinform* 2021;22:bbaa140. 10.1093/bib/bbaa140 32725161

[27] Zhao Y, Yin J, Zhang L. et al. Drug–drug interaction prediction: databases, web servers and computational models. *Brief Bioinform* 2024;25:bbad445. 10.1093/bib/bbad445 PMC10782925 38113076

[28] Han C-D, Wang C-C, Huang L. et al. MCFF-MTDDI: multi-channel feature fusion for multi-typed drug–drug interaction prediction. *Brief Bioinform* 2023;24:bbad215. 37291761 10.1093/bib/bbad215

[29] Li M, Wang Y, Li F. et al. A deep learning-based method for identification of bacteriophage-host interaction. *IEEE/ACM Trans Comput Biol Bioinform* 2021;18:1801–10. 10.1109/TCBB.2020.3017386 32813660 PMC8703204

[30] Li M, Zhang W. PHIAF: Prediction of phage-host interactions with GAN-based data augmentation and sequence-based feature fusion. *Brief Bioinform* 2022;23:bbab348. 34472593 10.1093/bib/bbab348

[31] Zhang Y-z, Liu Y, Bai Z. et al. Zero-shot-capable identification of phage–host relationships with whole-genome sequence representation by contrastive learning. *Brief Bioinform* 2023;24:bbad239. 10.1093/bib/bbad239 37466138 PMC10516345

[32] Shang J, Sun Y. Predicting the hosts of prokaryotic viruses using GCN-based semi-supervised learning. *BMC Biol* 2021;19:250. 10.1186/s12915-021-01180-4 34819064 PMC8611875

[33] Shang J, Sun Y. CHERRY: a computational method for accurate prediction of virus–prokaryotic interactions using a graph encoder–decoder model. *Brief Bioinform* 2022;23:bbac182. 10.1093/bib/bbac182 PMC9487644 35595715

[34] Ma L, Gao P, Zhou W. et al. Multi-view attention graph convolutional networks for the host prediction of phages. *Knowledge-Based Syst* 2025;308:112755. 10.1016/j.knosys.2024.112755

[35] Zhi-Hua D, Zhong J-P, Liu Y. et al. Prokaryotic virus host prediction with graph contrastive augmentaion. *PLoS Comput Biol* 2023;19:e1011671. 10.1371/journal.pcbi.1011671 38039280 PMC10691718

[36] Wang Y, Sun H, Wang H. et al. An effective model for predicting phage-host interactions via graph embedding representation learning with multi-head attention mechanism. *IEEE J Biomed Health Inform* 2023;27:3061–71. 10.1109/JBHI.2023.3261319 37030796

[37] Xiao Z, Sun H, Wei A. et al. A novel framework for predicting phage-host interactions via host specificity-aware graph autoencoder. *IEEE J Biomed Health Inform* 2025;29:3069–78. 10.1109/JBHI.2024.3500137 40030240

[38] Kauffman KM, Chang WK, Brown JM. et al. Resolving the structure of phage–bacteria interactions in the context of natural diversity. *Nat Commun* 2022;13:372. 10.1038/s41467-021-27583-z PMC8766483 35042853

[39] Alley EC, Khimulya G, Biswas S. et al. Unified rational protein engineering with sequence-based deep representation learning. *Nat Methods* 2019;16:1315–22. 10.1038/s41592-019-0598-1 31636460 PMC7067682

[40] LeCun Y, Bottou L, Bengio Y. et al. Gradient-based learning applied to document recognition. *Proc IEEE* 1998;86:2278–324. 10.1109/5.726791

[41] Chung J, Gulcehre C, Cho KH. et al. Empirical evaluation of gated recurrent neural networks on sequence modeling. arXiv:1412.3555. 2014. https://arxiv.org/abs/1412.3555

[42] Chawla NV, Bowyer KW, Hall LO. et al. SMOTE: synthetic minority over-sampling technique. *J Artif Intell Res* 2002;16:321–57.

[43] Li M, Liu G, Song W. et al. Enhancing strain-level phage-host prediction through experimentally validated negatives and feature optimization strategies. bioRxiv:2025.05.31.656987. 2025. 10.1101/2025.05.31.656987

[44] Coelho ED, Arrais JP, Matos S. et al. Computational prediction of the human-microbial oral interactome. *BMC Syst Biol* 2014;8:24. 10.1186/1752-0509-8-24 PMC3975954 24576332

[45] Boeckaerts D, Stock M, Criel B. et al. Predicting bacteriophage hosts based on sequences of annotated receptor-binding proteins. *Sci Rep* 2021;11:1467. 10.1038/s41598-021-81063-4 33446856 PMC7809048

[46] Nami Y, Imeni N, Panahi B. Application of machine learning in bacteriophage research. *BMC Microbiol* 2021;21:1–8. 10.1186/s12866-021-02256-5 34174831 PMC8235560

[47] Besemer J, Lomsadze A, Borodovsky M. GeneMarkS: a self-training method for prediction of gene starts in microbial genomes. Implications for finding sequence motifs in regulatory regions. *Nucleic Acids Res* 2001;29:2607–18. 10.1093/nar/29.12.2607 11410670 PMC55746

[48] Baker DN, Langmead B. Dashing: Fast and accurate genomic distances with hyperloglog. *Genome Biol* 2019;20:265. 31801633 10.1186/s13059-019-1875-0 PMC6892282

[49] Krause B, Murray I, Renals S. et al. Multiplicative LSTM for sequence modelling. In: 5th International Conference on Learning Representations Workshop. Toulon, France: OpenReview, 2016.

[50] Arif M, Ali F, Ahmad S. et al. Pred-BVP-Unb: fast prediction of bacteriophage virion proteins using un-biased multi-perspective properties with recursive feature elimination. *Genomics* 2020;112:1565–74. 10.1016/j.ygeno.2019.09.006 31526842

[51] Jing X-Y, Li F-M. Predicting cell wall lytic enzymes using combined features. *Front Bioeng Biotechnol* 2021;8:627335. 10.3389/fbioe.2020.627335 33585423 PMC7874139

[52] Srivastava N, Hinton G, Krizhevsky A. et al. Dropout: a simple way to prevent neural networks from overfitting. *J Mach Learn Res* 2014;15:1929–58.

[53] Alipanahi B, Delong A, Weirauch MT. et al. Predicting the sequence specificities of DNA-and RNA-binding proteins by deep learning. *Nat Biotechnol* 2015;33:831–8. 10.1038/nbt.3300 26213851

[54] Xie J, Jin X, Wei H. et al. IDP-EDL: enhancing intrinsically disordered protein prediction by combining protein language model and ensemble deep learning. *Brief Bioinform* 2025;26:bbaf182. 10.1093/bib/bbaf182 PMC12009716 40254833

[55] Reddi SJ, Kale S, Kumar S. On the convergence of Adam and beyond. In: 6th International Conference on Learning Representations. Vancouver, BC, Canada: OpenReview, 2018.

[56] Chang C-C, Lin C-J. LIBSVM: a library for support vector machines. *ACM Trans Intell Syst Technol* 2011;2:1–27. 10.1145/1961189.1961199

[57] Chen T, Guestrin C. XGBoost: a scalable tree boosting system. In: Proceedings of the 22nd ACM SIGKDD International Conference on Knowledge Discovery and Data Mining, pp. 785–94. San Francisco, CA, USA: ACM, 2016.

[58] Guo X, Chen F, Gao F. et al. CNSA: a data repository for archiving omics data. *Database* 2020;2020:baaa055. 32705130 10.1093/database/baaa055 PMC7377928

