# Supplementary material for: PBIP: a deep learning framework for predicting phage–bacterium interactions at the strain level
Source: Brief Bioinform. 2025 Dec 10;26(6):bbaf656. doi: 10.1093/bib/bbaf656 (PMC12694490; doi:10.1093/bib/bbaf656)
Supplement: Supplementary_Document_bbaf656 [file supplementary_document_bbaf656.pdf]

Supplementary Document of "PBIP: A Deep Learning Framework for Predicting Phage–Bacterium Interactions at the Strain Level"

Lijia Ma, Peng Gao, Gufeng Liu, Yuan Bai, Qiuzhen Lin, Jianqiang Li, and Minfeng Xiao

This is the supplementary document to the paper entitled "PBIP: A Deep Learning Framework for Predicting Phage–Bacterium Interactions at the Strain Level" and submitted to Briefings in Bioinformatics.

I. Detailed Acquisition Process of Protein Sequence Embedding

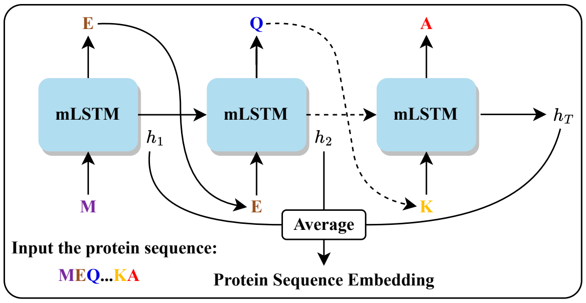

Fig. S1: Overview of generating a protein sequence embedding using UniRep.

Many methods for predicting PBIs rely on hand-crafted features, which often fail to capture complex biological patterns in protein sequences. To overcome this limitation, we employ UniRep [1], a pre-trained protein representation model that encodes sequences into 1,900-dimensional embeddings while preserving physico-chemical and structural properties. UniRep leverages a multiplicative LSTM (mLSTM) [2] trained to predict the next amino acid, thereby learning contextualized representations from raw sequences. The embedding process is illustrated in Fig. S1.

Formally, given a protein sequence of a phage or bacterium $S = (a_1, a_2, \ldots, a_T)$ with length T, where each element a_t $(1 \leq t \leq T)$ represents one of the twenty amino acids. First, the protein sequence S is one-hot encoded into a matrix $X_{\text{one}} \in \mathbb{R}^{T \times 20}$ as follows:

$$X_{\text{one}} = \text{OneHot}(S) = [E_1; E_2; \ldots; E_T], \tag{1}$$

where E_t is a binary vector of length 20 representing the amino acid a_t in the sequence S. The element E_{tj}, $1 \leq j \leq 20$, in the j-th position of E_t is set to 1 if it corresponds to a_t, and 0 otherwise.

Next, UniRep employs an embedding layer to convert the one-hot encoding matrix X_{one} into a continuous representation matrix $X_{\text{emb}} \in \mathbb{R}^{T \times 10}$. This embedding layer is parameterized by a weight matrix $W_{\text{emb}} \in \mathbb{R}^{20 \times 10}$, where each row corresponds to the continuous representation of an amino acid. In UniRep, W_{emb} is initialized with randomly generated values and is updated during training. Specifically, each one-hot vector E_t in X_{one} is replaced with its corresponding continuous vector from W_{emb}, as shown below:

$$X_{\text{emb}} = X_{\text{one}} W_{\text{emb}} = [X_1; X_2; \ldots; X_T], \tag{2}$$

where X_t represents the continuous embedding of the amino acid a_t.

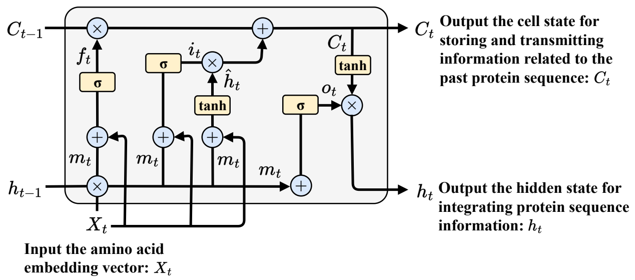

Fig. S2: The architecture of the mLSTM cell.

Following this, UniRep uses a multiplicative long short-term memory (mLSTM) network [2] with 1,900 hidden units to generate a compressed deep embedding of the protein sequence. At each time step t, the amino acid embedding X_t is fed into the mLSTM, which updates its hidden state h_t. The final sequence representation is obtained by averaging all hidden states across the sequence. The internal structure of the mLSTM is illustrated in Fig. S2.

The mLSTM introduces a multiplication operation to compute an intermediate state m_t, crucial for regulating protein information during the embedding compression process. This state is calculated as follows:

$$m_t = (W_m X_t) \odot (U_m h_{t-1}), \tag{3}$$

where h_{t-1} is the hidden state from the previous step ($h_0 = 0$), encapsulating prior sequence information. $\odot$ denotes element-wise multiplication. W_m and U_m are the weight matrices for X_t and h_{t-1} in the multiplication state, respectively.

The mLSTM also incorporates input, forget, and output gates to control the flow of information, enabling precise regulation of the sequence embedding.

The input gate helps the mLSTM determine whether the current embedding X_t contains relevant information that should

be integrated into the cell state. The input gate i_t is defined as:

$$i_t = \sigma(W_i X_t + U_i m_t), \tag{4}$$

where W_i and U_i are the weight matrices of X_t and m_t in the input gate, respectively, and σ is the sigmoid activation function.

The forget gate enables the mLSTM to decide which historical information in the cell state is irrelevant and can be discarded. The forget gate f_t is computed as:

$$f_t = \sigma(W_f X_t + U_f m_t), \tag{5}$$

where W_f and U_f are the weight matrices of X_t and m_t in the forget gate, respectively.

The output gate controls whether the current cell state information should influence the hidden state. The output gate o_t is expressed as:

$$o_t = \sigma(W_o X_t + U_o m_t), \tag{6}$$

where W_o and U_o are the weight matrices of X_t and m_t in the output gate, respectively.

Moreover, the mLSTM uses the cell state C_t as internal storage, which is updated through the gate mechanism. Specifically, the forget gate f_t is multiplied by the previous cell state C_{t-1} to retain necessary historical information. The input gate i_t is multiplied by the input candidate $\hat{h}_t$ to add new information. The cell state C_t is updated as follows:

$$\hat{h}_t = \tanh(W_h X_t + U_h m_t), \tag{7}$$

$$C_t = f_t \odot C_{t-1} + i_t \odot \hat{h}_t, \tag{8}$$

where W_h and U_h are the learned weight matrices of X_t and m_t in the candidate value, respectively. $\tanh$ is the hyperbolic tangent function.

Finally, the hidden state h_t abstracts and compresses the cell state to represent the sequence at each step. The hidden state h_t is computed as follows:

$$h_t = o_t \odot \tanh(C_t). \tag{9}$$

UniRep trains the mLSTM by predicting the next amino acid in the sequence and optimizes it using a cross-entropy loss function. During training, both the embedding layer weight matrix W_{emb} and the mLSTM parameters are jointly updated. The deep embedding representation x of the protein sequence S is obtained by averaging the hidden states as follows:

$$x = \frac{1}{T}\sum_{t=1}^{T} h_t. \tag{10}$$

We employ the officially released pre-trained UniRep weights (https://github.com/churchlab/UniRep), which include both W_{emb} and the mLSTM parameters. Using these pre-trained weights ensures the preservation of biologically meaningful features.

Since each phage or bacterium contains multiple proteins, we compute organism-level embeddings by averaging the corresponding protein embeddings. Specifically, we let x_{pi} and x_{bi} denote the UniRep embeddings of the i-th protein in a phage and bacterium, respectively. The organism-level protein embeddings are calculated as follows:

$$x_p = \frac{1}{N_p}\sum_{i=1}^{N_p} x_{pi}, \quad x_b = \frac{1}{N_b}\sum_{i=1}^{N_b} x_{bi}, \tag{11}$$

where N_p and N_b denote the number of protein sequences in the phage and bacterium. The combined embedding for a phage-bacterium pair $[P_k, B_k]$ is represented as $[x_p, x_b]_k$.

II. Detailed Technical Description of Bi-GRU Module

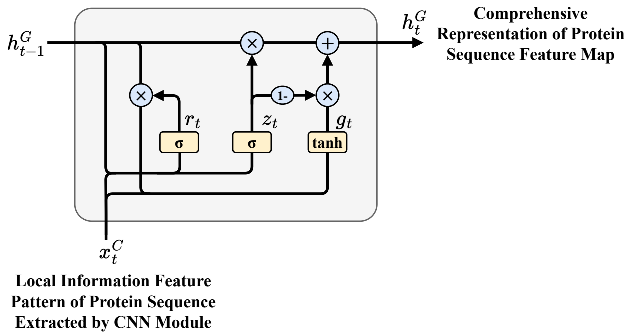

Fig. S3: The architecture of the GRU cell.

Following the convolutional neural network (CNN) module, a bi-directional gated recurrent unit (Bi-GRU) [3] is employed to capture long-term dependencies from both forward and backward directions. The internal structure of the GRU cell is presented in Fig. S3.

The local feature map $x^C = [x_1^C, x_2^C, \ldots, x_{N_f}^C]$ extracted by the CNN module is fed into the Bi-GRU module, where each feature pattern x_t^C is treated as a time step. The GRU cell utilizes only the update gate and the reset gate to regulate information flow.

The reset gate enables the GRU to reset some of the information in the hidden state h_{t-1}^G from the previous time step, effectively discarding protein sequence information that is weakly correlated with the current input. The reset gate r_t is defined as:

$$r_t = \sigma(W_r x_t^C + U_r h_{t-1}^G + b_r), \tag{12}$$

where W_r and U_r are the weights of x_t^C and h_{t-1}^G in the reset gate, respectively, while b_r is the bias term.

The update gate determines the extent to which the hidden state h_t^G at the current time step is derived from the previous hidden state h_{t-1}^G and the current memory content g_t, thus allowing the GRU to balance the contributions of the previous hidden state and the current inputs. The update gate z_t is computed as:

$$z_t = \sigma(W_z x_t^C + U_z h_{t-1}^G + b_z), \tag{13}$$

where W_z and U_z represent the weights of x_t^C and h_{t-1}^G in the update gate, while b_z is the bias term.

The GRU uses the memory content g_t to store both the previous information h_{t-1}^G and the current feature pattern x_t^C, effectively summarizing the current protein sequence information. The current memory content g_t is expressed as:

$$g_t = \tanh(W_g x_t^C + U_g(r_t \odot h_{t-1}^G) + b_g), \tag{14}$$

where W_g and U_g are the weights of x_t^C and $r_t \odot h_{t-1}^G$ in the current memory content, while b_g is the bias term.

Finally, the GRU produces the hidden state h_t^G, providing a comprehensive representation of the feature map that captures long-term dependencies between feature patterns. Specifically, when z_t approaches 1, the GRU will emphasize the previous hidden state h_{t-1}^G, whereas when z_t approaches 0, it will pay more attention to the current memory content g_t. The hidden state h_t^G is computed as follows:

$$h_t^G = (1 - z_t) \odot h_{t-1}^G + z_t \odot g_t. \tag{15}$$

Compared to the GRU, the Bi-GRU processes the protein feature map in both forward and backward directions, enabling it to capture long-term dependencies and contextual information in the protein sequence. The final hidden state h_t^G is computed as:

$$h_t^G = \overrightarrow{h_t^G} \oplus \overleftarrow{h_t^G}, \tag{16}$$

where $\overrightarrow{h_t^G}$ and $\overleftarrow{h_t^G}$ represent the forward and backward hidden states, respectively, and $\oplus$ denotes the concatenation operation.

References

[1] E. C. Alley, G. Khimulya, S. Biswas, M. AlQuraishi, and G. M. Church, "Unified rational protein engineering with sequence-based deep representation learning," *Nature methods*, vol. 16, no. 12, pp. 1315–1322, 2019.

[2] B. Krause, L. Lu, I. Murray, and S. Renals, "Multiplicative lstm for sequence modelling," *arXiv preprint arXiv:1609.07959*, 2016.

[3] J. Chung, C. Gulcehre, K. Cho, and Y. Bengio, "Empirical evaluation of gated recurrent neural networks on sequence modeling," *arXiv preprint arXiv:1412.3555*, 2014.
